# An Eco-Friendly Conversion of Aquaculture Suspended Solid Wastes Into High-Quality Fish Food by Improving Poly-β-Hydroxybutyrate Production

**DOI:** 10.3389/fphys.2022.797625

**Published:** 2022-05-26

**Authors:** Guo Qiao, Xiaoxia Li, Jun Li, Mingming Zhang, Yang Shen, Zhigang Zhao, Yichan Zhang, Zhitao Qi, Peng Chen, Yuyu Sun, Pingping Cang, Peng Liu, Eakapol Wangkahart, Zisheng Wang

**Affiliations:** ^1^ Yancheng Institute of Technology, Yancheng, China; ^2^ School of Biotechnology, Jiangsu University of Science and Technology, Zhenjiang, China; ^3^ Department of Biological Sciences, Biomolecular Sciences Institute, Florida International University, Miami, FL, United States; ^4^ Heilongjiang Provincial Key Laboratory of Cold Water Fish Germplasm Resources and Aquaculture, Heilongjiang River Fisheries Research Institute, Chinese Academy of Fishery Sciences, Harbin, China; ^5^ Yantai Marine Economic Research Institute, Yantai, China; ^6^ Laboratory of Fish Immunology and Nutrigenomics, Applied Animal and Aquatic Sciences Research Unit, Division of Fisheries, Faculty of Technology, Mahasarakham University, Maha Sarakham, Thailand

**Keywords:** aquaculture solid wastes, nitrogenous compounds, biopolymer, poly-β-hydroxybutyrate (PHB), accumulation optimization, fish food

## Abstract

The aquaculture industry is vital in providing a valuable protein food source for humans, but generates a huge amount of solid and dissolved wastes that pose great risks to the environment and aquaculture sustainability. Suspended solids (in short SS), one of the aquaculture wastes, are very difficult to be treated due to their high organic contents. The bioconversion from wastewater, food effluents, and activated sludge into poly-β-hydroxybutyrate (PHB) is a sustainable alternative to generate an additional income and could be highly attractive to the agricultural and environmental management firms. However, little is known about its potential application in aquaculture wastes. In the present study, we first determined that 7.2% of SS was PHB. Then, the production of PHB was increased two-fold by the optimal fermentation conditions of wheat bran and microbial cocktails at a C/N ratio of 12. Also, the PHB-enriched SS showed a higher total ammonia nitrogen removal rate. Importantly, we further demonstrated that the PHB-enriched SS as a feed could promote fish growth and up-regulate the expression of the immune-related genes. Our study developed an eco-friendly and simple approach to transforming problematic SS wastes into PHB-enriched high-quality food for omnivorous fish, which will increase the usage efficiency of SS and provide a cheaper diet for aquatic animals.

## 1 Introduction

The aquaculture industry provides human society with a nutritional food source and high proteins, vitamins, and macro minerals (Henriksson et al., 2018; Han et al., 2019; Heiderscheidt et al., 2020). During the rapid development of the aquaculture industry, two problems have become increasingly apparent: 1) feeding is expensive and 2) a large amount of waste and discharge is generated. The aquaculture waste, containing high nitrogen and phosphorus, poses enormous environmental risks. In Japan, for example, one ton of pond-raised aqua-fish can generate 0.1 kg phosphorus and 0.8 kg nitrogen which equals to the daily waste produced by 73 people (Bangar et al., 2017). Therefore, aquaculture waste has raised the concerns of environmental scientists and challenges the sustainable development of the aquaculture (Ahmed et al., 2019; Han et al., 2019). Many countries have established standards for wastewater discharge, and strictly require that aquaculture wastes can be emitted into the environment only within the allowable range.

The byproducts of feeding and excretion, such as unconsumed food, undigested components, feces, chemicals, and untapped inputs, are the sources of aquaculture wastes (Kokou & Fountoulaki, 2018; Becke et al., 2020; Gibson et al., 2020). The wastes from aquaculture can be divided into solid wastes and suspended wastes, both contain high organic contents (Olusegun et al., 2016; Gao et al., 2020; Hesni et al., 2020; Naughton et al., 2020; Rosa et al., 2020; Sun et al., 2020). The solid wastes include the settled solids sunk to the bottom of the pond and the suspended solids (in short SS) floating in the culture water. In the traditional aquaculture system, the SS are the majority of waste products, and are challenging to be removed due to their high organic contents (Badiola et al., 2012; Becke et al., 2020). However, the SS must be properly managed to avoid the pollution to the surrounding environment and reduce the harm to aquatic animals (Holm-Nielsen et al., 2009; Kiani et al., 2020). Notably, SS can be fermented by bacteria to remove toxic organic contents for resource recycling (Ge and Champagne, 2017; Das et al., 2018; Amadu et al., 2021). These fast-growing microbes will change the toxic ammonia (NH_3_) and nitrite (NO_2_^−^) into non-toxic nitrate (NO_3_^−^) (Lananan et al., 2014; Rommozzi et al., 2020). These ammonium ions and NH_3_ are produced during aquaculture (Karri et al., 2018; Yun et al., 2019).

The treatments about promoting microbial growth have been successfully applied in wastewater treatment (Ge et al., 2014; Qiu et al., 2020a; Xu M. et al., 2020; Xu X. et al., 2020). It can remove nitrogen and phosphorus from the aquatic environment (Qiu et al., 2020b; Xu M. et al., 2020; Xu X. et al., 2020). It also promotes microbial growth to produce valuable energy alternatives such as bio-fuel, natural antioxidants, food additives, or biopolymers (Meixner et al., 2017; Ge et al., 2018; Jochum et al., 2018; Qiu et al., 2020b). Among these alternatives, popular food additives and biopolymers such as poly-β-hydroxybutyrate (PHB) or polyhydroxyalkanoates (PHAs) are the most significant compounds that have been studied (Amadu et al., 2021). PHB or PHAs are not soluble in water. As intracellular energy storage, they form “inclusion bodies” within the cytoplasm in prokaryotic organisms. The chain of PHB is shorter than that of PHAs (Carpine et al., 2018). PHB is biodegradable, biocompatible, and nontoxic (Hrabak, 1992; Arun et al., 2006; Ben Rebah et al., 2007). A considerable effort has been made to produce PHB or PHAs using various wastes, such as municipal wastewater (Chua et al., 2003), sugar cane molasses (Albuquerque et al., 2010), paper mill wastewater (Bengtsson et al., 2008), and food waste (Venkateswar Reddy et al., 2015). However, transforming aquaculture wastes into PHAs or PHB has not been well-investigated (Krasaesueb et al., 2019).

The biofloc technology is a new strategy to reduce the total ammonia nitrogen (TAN) effectively through heterotrophic microbiota by adjusting the C/N ratio in the culture water via external carbon addition (Avnimelech, 1999; Zhang et al., 2018). It was reported that SS in the biofloc system contains 15–20% PHB (Schryver & Verstraete, 2009; Ruan et al., 2011). The commercial PHB has been used as a dietary supplement to improve the growth, immunity, and disease resistance of aquatic animals (Deng et al., 2014; Defoirdt et al., 2018; Meirong et al., 2018; Qiao et al., 2018, 2020). However, the high prices for commercial PHB and water-insolubility greatly hinder its application in aquaculture.

In this study, we analyzed the PHB content in SS waste from a traditional aquaculture system, and tried to increase the PHB production by adjusting the carbon source, various C/N ratios, and bacterial strains. Furthermore, we evaluated the TAN removal during the PHB-enrichment process, analyzed the effects of PHB-enriched SS on the growth, and innate immunity of an omnivorous fish—gibel carp (*Carassius auratus gibelio*). This study will provide a good low-cost method to treat and recycle aquaculture solid wastes, and present a potential high-quality fish food and convenient method for PHB application in aquaculture.

## 2 Materials and Methods

### 2.1 Suspended Solid Collection and Analysis

Three indoor concrete tanks cultured gibel carps (40.33 ± 5.12 g) at the Yancheng Institute of Technology were randomly selected to acquire SS. Gibel carp was fed with commercial diets (Tongwei Feeding Company, China) to satiation three times (6:30 to 7:00 a.m., 1:30 p.m. to 2:00 p.m., and 7:00 p.m. to 7:30 p.m.) each day under a 12D/12L cycle. The diets contained 32.25% crude protein, 5.90% crude lipid, 1.18% calcium, and 1.23% total phosphorus. Water temperature was maintained at 25.0 ± 2.0°C and dissolved oxygen (DO) kept higher than 5 mg L^−1^. SS were sampled *in situ* using the Imhoff cone, that is, culture water from three tanks was withdrawn from the middle of tank into the Imhoff cone. Then, SS were collected from the bottom of the Imhoff cone (Zhang et al., 2018). The obtained SS were used to analyze the basic characterization according to the standard method (APHA, 2012; Krasaesueb et al., 2019), including pH, temperature, concentration of nitrate (NO_3_^−^–N), nitrite (NO_2_^−^–N), ammonium (NH_4_^+^–N), phosphate (PO_4_^3-^–P), and total phosphorus. Finally, we removed water- and acetone-soluble materials with the following steps: 1) collecting pellets by centrifugation of the SS at 8,000 rpm for 10 min, 2) resuspending the pellet in acetone (2V), 3) collecting pellets again by centrifugation of the SS at 8,000 rpm for 10 min, 4) washing with sterile distilled water, and 5) freeze-drying the pellets to check the PHB content as described below.

### 2.2 Poly-β-Hydroxybutyrate Content and Characterization in Suspended Solids

#### 2.2.1 Crude Poly-β-Hydroxybutyrate Extraction

The PHB was extracted according to the procedure described by Ruan et al. (2011) with some modifications. In brief, the freeze-dried SS were dissolved in 10% NaClO and disrupted by ultrasonic for 8 min (intermittent 5 s, working 5 s, 4°C), and the precipitate was obtained by centrifugation at 8,000 rpm for 15 min at room temperature. Then, the pellet was washed twice with acetone and sterile distilled water to remove lipids by centrifugation at 8,000 rpm for 10 min. Finally, the crude PHB was obtained after the pellet dried at 50°C for 8 h in an oven.

#### 2.2.2 Poly-β-Hydroxybutyrate Content Analysis

The crude PHB was diluted with chloroform at 1:40 (dry weight of SS:volume of chloroform, g:mL) at 40°C for 11 h, and the pellet was collected by centrifugation, dried at 70°C for 8 h in the oven. Then, the PHB extract was diluted with chloroform at 80°C using a rotary evaporator (Eyela N-1000), and dried to a constant mass. A thin film of pure PHB was obtained and quantified using spectrometric analysis based on the standard curve (Law & Slepecky, 1961). The purity of PHB was analyzed by gas chromatography (GC–MS, Agilent 7890B/5977A) (Albuquerque et al., 2010; Alsafadi & Al-Mashaqbeh, 2017).

#### 2.2.3 Poly-β-Hydroxybutyrate Characterization

The structural and material properties of the pure PHB were compared to a commercial PHB (Aldrich, Sigma). The surface morphology and elements of the pure PHB were observed by the scanning electron microscopy (SEM–EDS). The Fourier-transform infrared (FT-IR) spectra of the pure PHB extract were detected in the range of 400–4,000 cm^−1^ using a spectrophotometer (FT-IR Thermo is 10) (Naumann et al., 1991; Venkateswar Reddy et al., 2015).

### 2.3 Bacterial Community Analysis With Poly-β-Hydroxybutyrate Accumulation in Suspended Solids

#### 2.3.1 DNA Extraction and PCR Amplification

Since PHB is synthesized by bacteria and the yield correlates with bacterial diversity closely (Crab et al., 2012; Qiao et al., 2018; Amadu et al., 2021), the bacterial community in SS was investigated by high-throughput sequencing using a MiSeq sequencing platform. In brief, the quadruple SS from each tank were sampled, immediately frozen in liquid nitrogen, and then transferred to −80°C for DNA extraction. Microbial DNA from the SS was extracted using the E.Z.N.A.^®^ soil DNA kit (OMEGA, United States) following the manufacturer’s protocol. PCR was used to amplify the V4–V5 region through primers 907R (5′-CCGTCAATTCMTTTRAGTT T-3′) and 515F (5′-barcode-GTGCCAGCMGCCGCGG-3′) (Qiao et al., 2019b). PCR products were purified using the AxyPrep DNA gel extraction kit (Axygen Biosciences, Union City, CA, United States).

#### 2.3.2 Illumina MiSeq Sequencing

The purified PCR products were first quantified using Qubit^®^3.0 (Life Invitrogen). About 24 amplicons with different barcodes were mixed equally, and the pooled products were chosen to construct an Illumina Paired–End library according to the Illumina’s genomic DNA library preparation procedure. Finally, the amplicon library was paired-end sequenced (2 × 250) on the MiSeq sequencing platform (Illumina, United States) according to the standard protocols (Sangon Biotech (Shanghai) Co., Ltd.).

#### 2.3.3 Bioinformatics Analysis

The bioinformatics analysis from Illumina sequencing was constructed using the methods described previously (Qiao et al., 2019b). In brief, QIIME (version 1.17) was used to de-multiplex and quality-filter the raw FASTQ files, and any unassembled reads were discarded. Bacterial operational taxonomic units (OTUs) were generated using the uclust function in QIIME (http://qiime.org/scripts/pick.outs.html). The Shannon index calculated by mothur (version v.1.30.1) was analyzed to illustrate α-diversity. The OTUs were mapped to a gg13.5 database by QIIME’s command “pick_ closed_otus” at 97% similarity. The OTU abundance was automatically normalized using 16S rRNA gene copy numbers from known bacterial genomes in Integrated Microbial Genomes.

### 2.4 Accumulation Optimization of Poly-β-Hydroxybutyrate in Suspended Solids

PHB as the carbon and energy reserves is stored in bacterial cells at the appropriate C/N ratio. PHB production is related to the C/N ratio, carbon sources, and bacterial community (Crab et al., 2012; Qiao et al., 2018; Amadu et al., 2021). Based on the most practical usage of carbon sources and probiotics, and the C/N ratio in aquaculture (Laranja et al., 2018), we designed various C/N ratios, carbon sources, and additional bacteria to study parameters that affect the yield of PHB. The detailed protocols are: 1) SS were collected, 2) then diluted into sterilized freshwater, 3) different C/N ratios (12, 16, and 20) using molasses as the sole carbon resource were set to illustrate the effect of the C/N ratio on PHB accumulation, 4) different carbon sources (molasses, starch, wheat bran, and corn meal) and bacteria (*Bacillus subtilis* SY01, *Pseudomonas putida* CGMCC15104 and *Lactobacillus*, and a cocktail of *Pse. putida* and *Lactobacillus*) were separately added based on the total ammonia concentration by adapting the C/N ratio of 12 each day for 7 days, and 5) PHB accumulation was evaluated. *Pse. putida* CGMCC15104 and *Lactobacillus* were obtained as described previously (Xu, 2019), and *B. subtilis* SY01 and *Pse. putida* were isolated from SS in our laboratory (Xu, 2019). During the SS treatment, the usage dose of carbon sources was calculated according to the TAN concentration and kept the C/N ratio of 12. The carbon sources were diluted with water and sprinkled in the water twice every 12 h within 1 day. The final bacterial concentration of addition was 1.0 × 10^7^ CFU mL^−1^. All the experimental groups were conducted in triplicates.

The relative production rate of PHB was calculated according to Eq. 1.

$$\text{Relative}\;\text{production}\;\text{rate}\left(\text{RPR},\text{\%}\right)=\frac{\text{PR}\;\text{in}\;\text{different}\;\text{treated}\;\text{groups }}{\text{PR}\;\text{in}\;\text{untreated}\;\text{SS}\;\text{group}}\times 100.$$ (1)

### 2.5 Benefit Evaluation of Poly-β-Hydroxybutyrate-Enriched Suspended Solid Treatment in the Aquaculture System

#### 2.5.1 Poly-β-Hydroxybutyrate-Enriched Suspended Solid Treatment

Based on the results from part 2.4 (Figure 6), PHB-enriched SS were obtained by adjusting the C/N ratio, carbon source, and probiotics addition. In brief, SS were randomly collected from three indoor concrete tanks and three outdoor ponds, which cultured fish and shrimp (Table 2). Then, the collected SS were diluted in sterilized freshwater at 1:10 (dry weight of SS:volume of freshwater). These SS were treated and kept at a C/N ratio of 12 by the addition of wheat bran twice every 12 h within 1 day under 24 h of continuous aeration for 7 days at pH 8.0 and temperature 25°C. Meanwhile, the cocktail of *Pseudomonas* and *Lactobacillus* was added at day 1 and day 3.

#### 2.5.2 Effects of Suspended Solid Treatment on the Removal Rate of Total Ammonia Nitrogen

At day 0 and day 7, the TAN concentration in the supernatant was measured according to the indophenol method of Koroleff (1976). The removal percentage of TAN from SS solutions was calculated according to Eq. 2.

$$Removal\;\left(\%\right)=\frac{\left(Ic\;-\;Fc\right)}{Ic\times \;100}$$ (2)

where *Ic* is the initial concentration (mg L^−1^) of TAN (day 0), and *Fc* is the final concentration (mg L^−1^) (day 7).

#### 2.5.3 The Nutritional Composition of Suspended Solids and Their Effects on the Growth Performance of Gibel Carp

##### 2.5.3.1 Nutritional Composition Analysis of Suspended Solids

In order to test whether SS could be up-taken by fish, and determine the composition of SS that affects fish production, we first detected the nutritional composition of SS. The nutritional composition was analyzed as our previous description (Zhang et al., 2018). In brief, basal SS were collected using the Imhoff cone and dried in an oven at 105°C to constant weight or freeze-dried to analyze the proximate composition, including the crude protein, lipid, ash, and total amino acid content (AOAC, 1995). The PHB content in the PHB-enriched SS and untreated SS was analyzed as described in Section 2.2.2.

##### 2.5.3.2 Effects of Suspended Solids on the Growth Performance of Gibel Carp

Gibel carp is a main ornamental freshwater culture species, and it can uptake SS in water as food sources (Zhang et al., 2018). Thus, in this study, gibel carp was chosen as a representative ornamental species to evaluate the potential usage of treated SS in aquaculture. Gibel carp (mean body weight of 12.03 g) was bought from a fish farm at Dafeng, Jiangsu province. Prior to the experiments, fish were acclimated for 2 weeks in a tank with continuous aeration at water pH of 7.4–8.2, and temperature of 25–27°C under a 12D/12L cycle. Fish were fed with a commercial diet (Tongwei, China) three times daily at 3% of their body weight, and 30% of water was exchanged. After acclimation, 30 individuals were sampled and separately weighed. Other fish were then randomly distributed into the experimental tanks.

**FIGURE 1:**
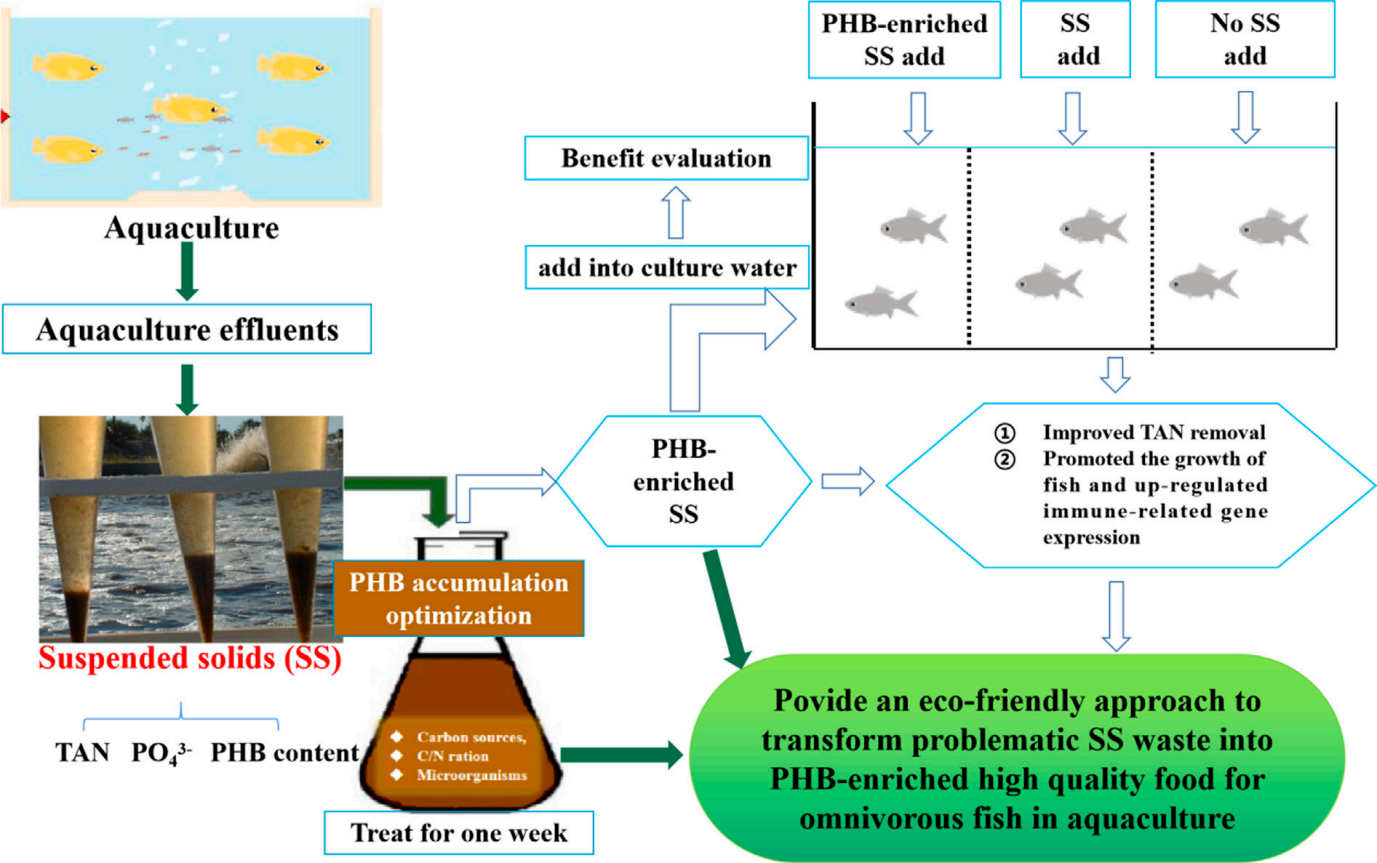
Graphical representation of the aquaculture suspended solid (SS) treatment process by adding carbons considering PHB accumulation.

Three groups were set, including the PHB-enriched SS addition (SSA), untreated SS addition (USSA), and no SS addition (NSSA) groups (Figure 1). The PHB content in PHB-enriched SS and untreated SS was 17.34 ± 4.76% and 7.06 ± 1.76%, respectively (Supplementary Table S2). Each group was housed in quadruplicate plastic tanks (size: 60 cm × 50 cm × 40 cm) for 30 days, and each tank included 20 fish. None of the fish in these three groups were fed with a commercial diet in order to test whether SS could be up-taken by fish, and further ensure positive effects of PHB in SS, that is, treated SS could potentially be used as feed. Total suspended solid (TSS) concentration is a good indicator for evaluating suspended solids. Based on the previous study (Zhang et al., 2018), the optimal TSS concentration for gibel carp was 600–800 mg L^−1^. Thus, TSS concentration in PHB-enriched SSA and USSA groups of the present study was kept at 600 mg L^−1^ through mixing aerated freshwater and stock SS water as previously described by Zhang et al. (2018), and TSS concentration was set at 10 mg L^−1^ (close to TSS concentration in natural freshwater) in the NSSA group by daily water-exchange. The TSS concentration was determined according to the Standard Methods for Examination of Water and Wastewater (APHA, 2012). During 30 days of feeding experiment, all fish were cultured at pH of 7.4–8.2, and a temperature of 25 ± 2°C under a 12D/12L cycle. The dead individuals would be cleaned up immediately.

At 30 days, all the fish from each group were weighed to calculate the growth parameters, including weight gain (WGR), specific growth rate (SGR), and thermal growth coefficient (TGC) according to Eqs 3–5.

$$\text{WGR }\left(\text{\%}\right)=\frac{100\times \left[\text{FW}\left(\text{g}\right)-\text{IW}\left(\text{g}\right)\right]}{FW},$$ (3)

$$\text{SGR}\left(\text{\%}\;\text{day}-1\right)=\frac{100\times \left(\text{LnFW}-\text{LnIW}\right)}{\text{time }\left(\text{days}\right)},$$ (4)

$$\text{TGC }=\;\left[\left(\sqrt[{3}]{\text{FW}}-\sqrt[{3}]{\text{IW}}\right)\mathrm{/Txt}\right]\times 1000,$$ (5)

where FW and IW are the final and initial body weight, respectively, T is duration of the experiment in days, and t is the mean daily water temperature.

##### 2.5.3.3 Effects of Suspended Solid Treatment on the Immune-Related Gene Expression of Gibel Carp

At day 30, five fish were sampled from each group, and anesthetized with tricaine methane sulfonate (MS-222) at 200 mg L^−1^ for immune-related gene transcription analysis. The spleen and gill (50–100 mg each tissue) were sampled, immediately immersed in 500 μL RNAiso Plus (Sigma), and stored at −80°C until total RNA extraction. RNeasy mini kit (Qiagen, Valencia, CA, United States) was used to extract total RNA (Zhang et al., 2017; Qiao et al., 2019a), and the quantity and purity of RNA were analyzed using a Nanodrop ND-1000 spectrophotometer. First-strand cDNA synthesis was conducted using the PrimeScript™ first-strand cDNA Synthesis kit (Takara Bio, Dalian, China) and the Oligo^dT^Primer. Six immune-related genes, including heat shock protein 70 (*hsp70*), tyrosine-protein kinase (*JAK*), interleukin-11 (*IL-11*), serine/threonine-protein kinase mTOR (*mTOR*), phosphatidylinositol 3-kinase regulatory subunit alpha (*PIK3R1*), and intelectin (*ITLN*), were selected to evaluate the different treatment of SS on the immune-related gene expression of gibel carp. The gene-specific primer list is shown in Supplementary Table S1, and quantitative real time PCR (qRT-PCR) performance was conducted as described in our previous publications (Zhang et al., 2018; Qiao et al., 2020). The relative transcriptional levels of different genes were determined using the following formula: ΔCt = Ct (target) - Ct (internal). The relative fold changes of a specific gene in fish from the PHB-enriched SSA and USSA groups were compared to those from the NSSA group using the 2^−ΔΔCt^ method (Livak & Schmittgen, 2001).

### 2.6 Data Processing and Statistical Analysis

All data are represented as mean ± standard deviation from triplicated or quadruple samples. Statistical analysis was conducted by Duncan’s multiple range test using one-way ANOVA with SPSS software (version 24.0). The significant difference was set at *p* value less than 0.05.

## 3 Results

### 3.1 Characterization of Suspended Solids

The pH of the collected SS was 8.12 ± 0.05, and the temperature was 27.20 ± 0.26°C. The concentrations of NO_3_^−^–N, NO_2_^−^–N, NH_4_^+^–N, TAN, PO_4_^3-^–P, and TP in SS were 14.57 ± 0.91 mg L^−1^, 1.88 ± 0.03 mg L^−1^, 3.15 ± 0.48 mg L^−1^, 19.6 ± 1.37 mg L^−1^, 9.45 ± 1.04 mg L^−1^, and 11.10 ± 0.40 mg L^−1^, respectively. The content of PHB in SS was 7.27 ± 0.91% (w/w dry weight) (Table 1). Apparently, nitrogen and phosphorus are the dominant elements in SS.

**TABLE 1:** Characterization of basal suspended solids (SS) collected from aquaculture system.

Parameters	Initial SS from indoor concrete tank (gibel carp)	Unit
pH	8.12±0.05	-
Temperature	27.20±0.26	°C
Phosphate (PO_4_^3−P^)	9.45±1.04	mg L^−1^
Nitrate (NO_3_^−N^)	14.57±0.91	mg L^−1^
Nitrite (NO_2_^−N^)	1.88±0.03	mg L^−1^
Ammonium (NH_4_^+−N^)	3.15±0.48	mg L^−1^
Total ammonia nitrogen (TAN)	19.60±1.37	mg L^−1^
Total phosphorus (TP)	11.10±0.40	mg L^−1^
PHB content	7.27±0.91	% (w/w dry weight)

PHB, poly-β-hydroxybutyrate.

### 3.2 Polymer Structure and Material Properties of Poly-β-Hydroxybutyrate in Suspended Solids

**FIGURE 2:**
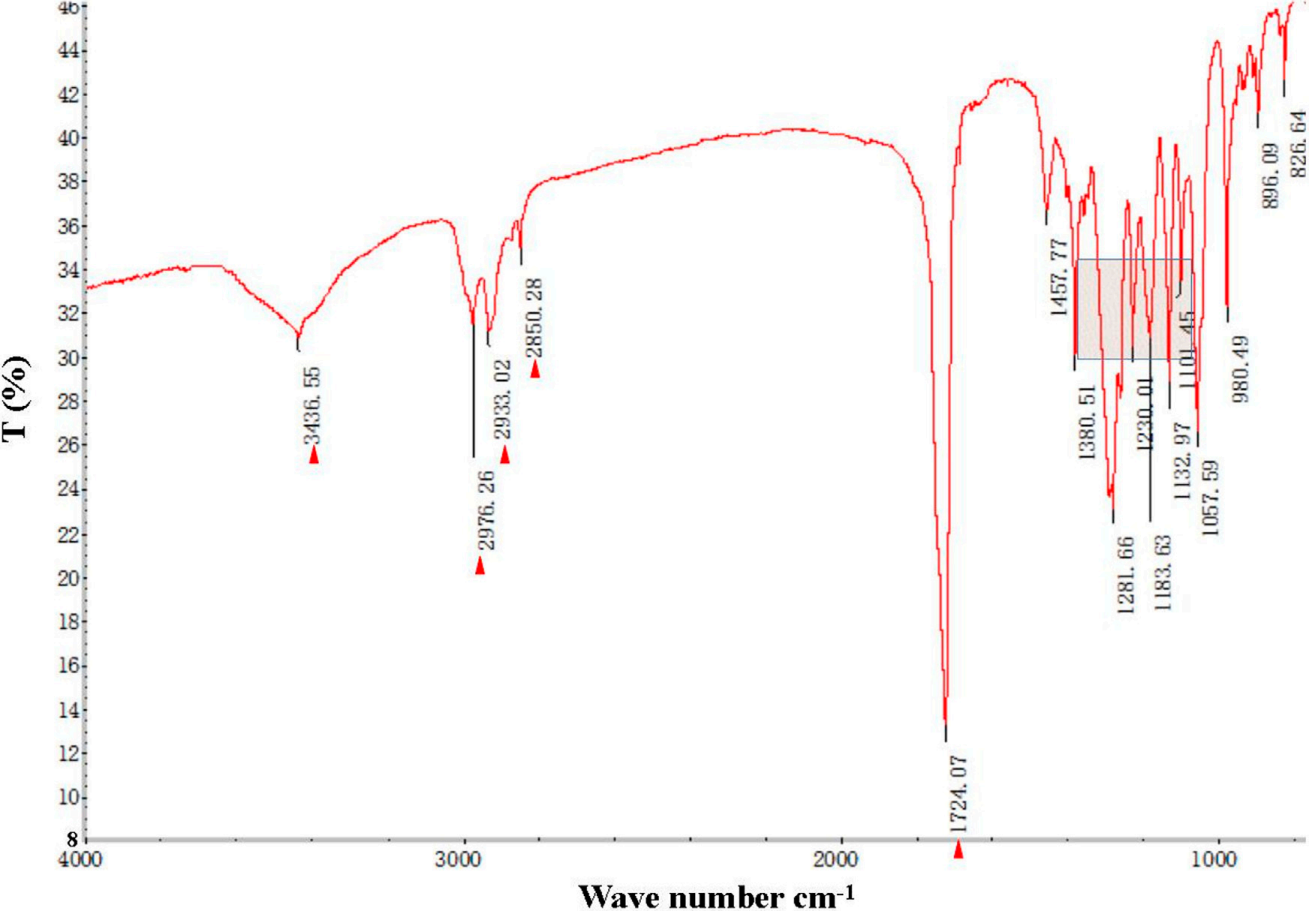
Fourier transform infrared (FT-IR) spectra of PHB extract from SS. Red triangle and box with blue line mean bands of PHB.

**FIGURE 3:**
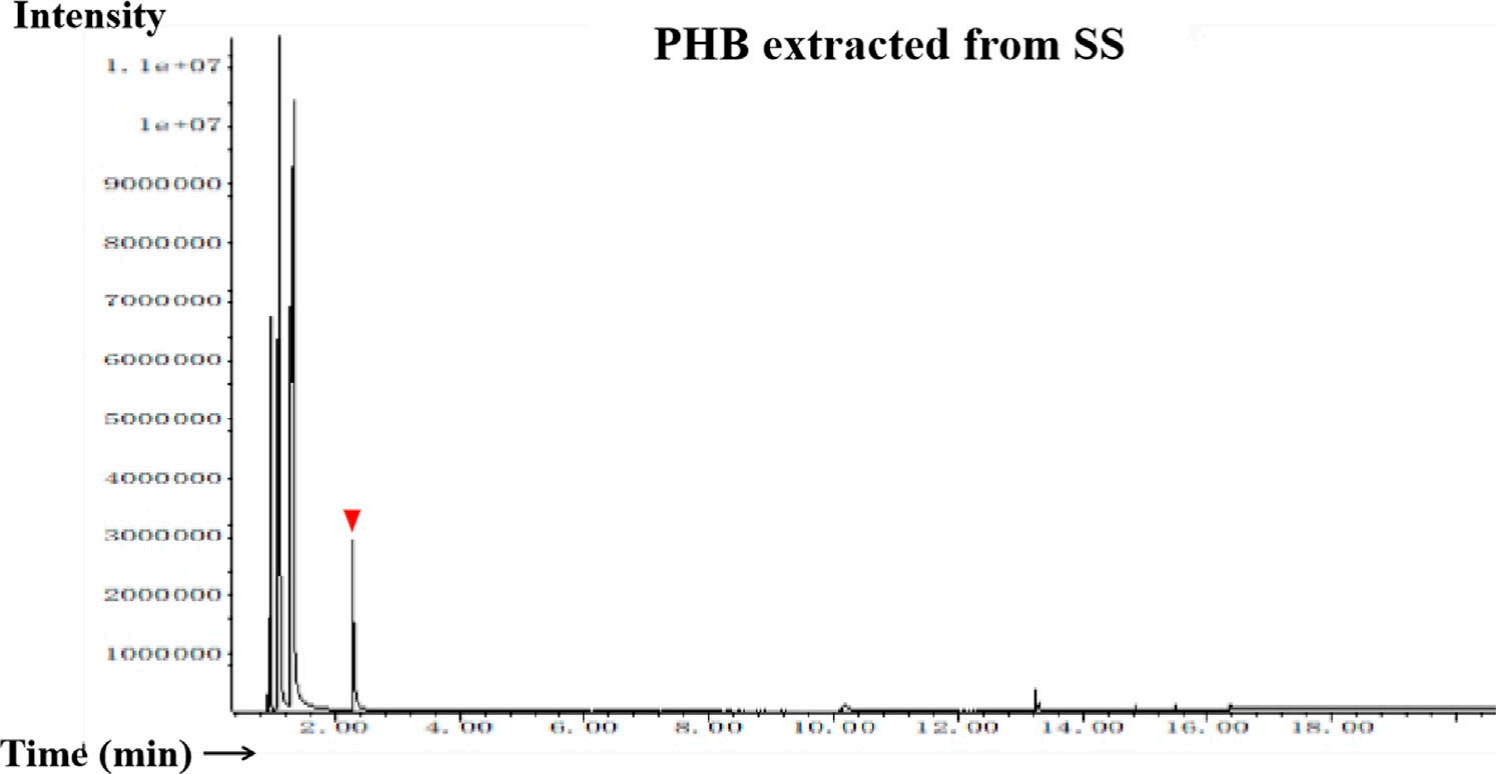
Gas chromatogram of PHB extract from SS. Red triangle means the retention time.

**FIGURE 4:**
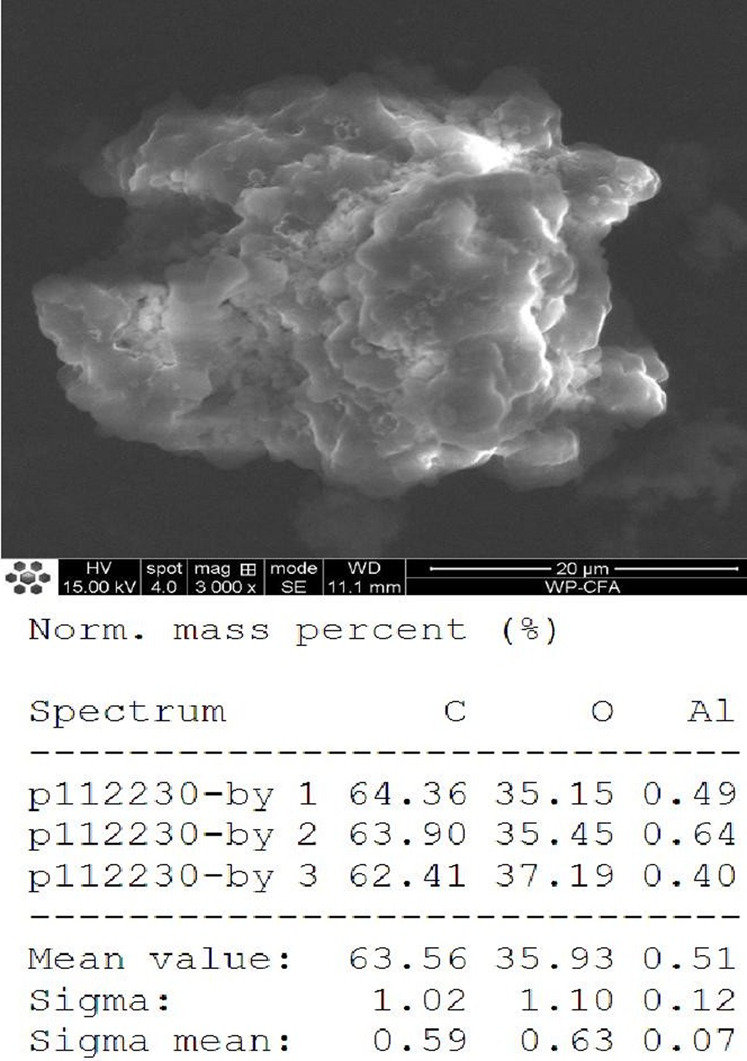
Scanning electron microscopy observation of PHB extract from SS.

The FT-IR spectra of the extracted PHB were measured to confirm the practical structure (Figure 2). The band at 1724 cm^−1^ corresponded to the stretching of the C=O bond, whereas a series of intense bands located at 980–1,457 cm^−1^ corresponded to the stretching of the C–O bond of the ester group. The methylene C–H stretching vibration near 2,933 cm^−1^ was also observed. The presence of absorption bands at 1724 cm^−1^ and 1,281 cm^−1^ in the extracted PHB sample were characteristics of C=O and C–O stretching groups, and identical to standard PHB. GC–MS analysis showed that the retention time of the extracted PHB sample was 2.214 min (Figure 3). SEM–EDS analysis showed that the content of elements C and O in the PHB extract from SS was 63.56% of carbon and 35.93% of oxygen, respectively (Figure 4). These data confirmed that our extract PHB is the same as the standard PHB.

### 3.3 Bacterial Diversity Related to Poly-β-Hydroxybutyrate Accumulation in Suspended Solids

We analyzed the bacterial diversity in SS (Supplementary Figure S1). The plateau OTU rarefaction curve after the similarity cutoff of 97% indicated that the sequencing data were reasonable. Furthermore, the Shannon index and relative abundance suggested that the sequences obtained can represent most bacteria in each sample.

**FIGURE 5:**
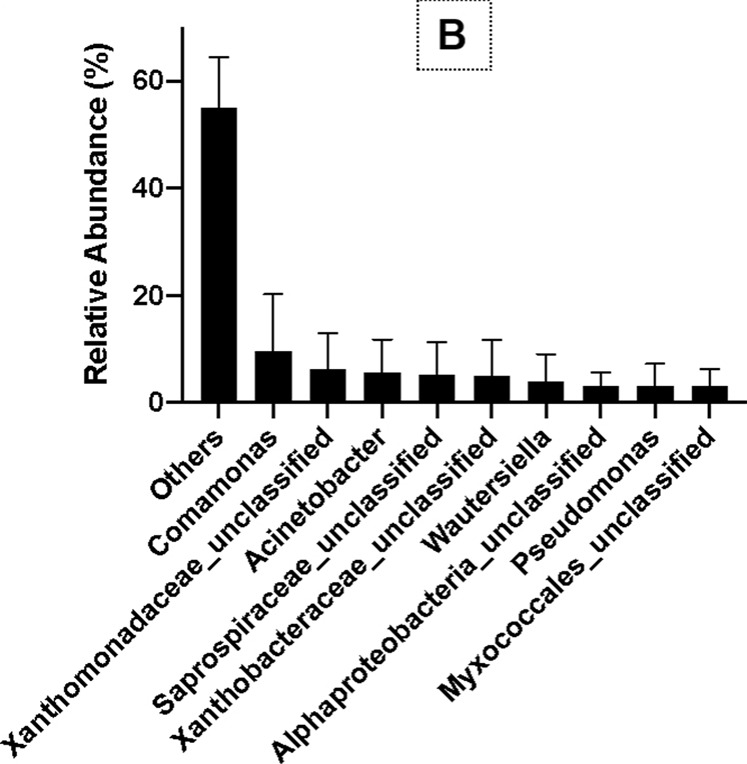
Bacterial diversity at the class level **(A)** and the genus level **(B)** in SS by MiSeq sequencing.

The results of OTU analysis using 16S rRNA gene copy numbers from known bacterial genomes showed that the relative abundance of ɤ-Proteobacteria, β-Proteobacteria, α-Proteobacteria, Saprospirae, Flavobacteria, Deltaproteobacteria, Cytophagia, and Fusobacteria was 24%, 21%, 19%, 7%, 7%, 2%, 2%, and 1%, respectively, at the class level (Figure 5A). At the family and genus levels, the relative abundance of *Comamonas*, Xanthomonadaceae_unclassified, *Acinetobacter*, Saprospiraceae_ unclassified, Xanthobacteraceae_unclassified, *Wautersiella*, *Pseudomonas*, and Myxococcales_unclassified were 9%, 6%, 5%, 5%, 5%, 4%, 3%, and 3%, respectively (Figure 5B). Thus, *Comamonas*, *Acinetobacter*, and *Pseudomonas* are the most dominant bacteria related to PHB production in SS.

### 3.4 Poly-β-Hydroxybutyrate Accumulation Optimization

**FIGURE 6:**
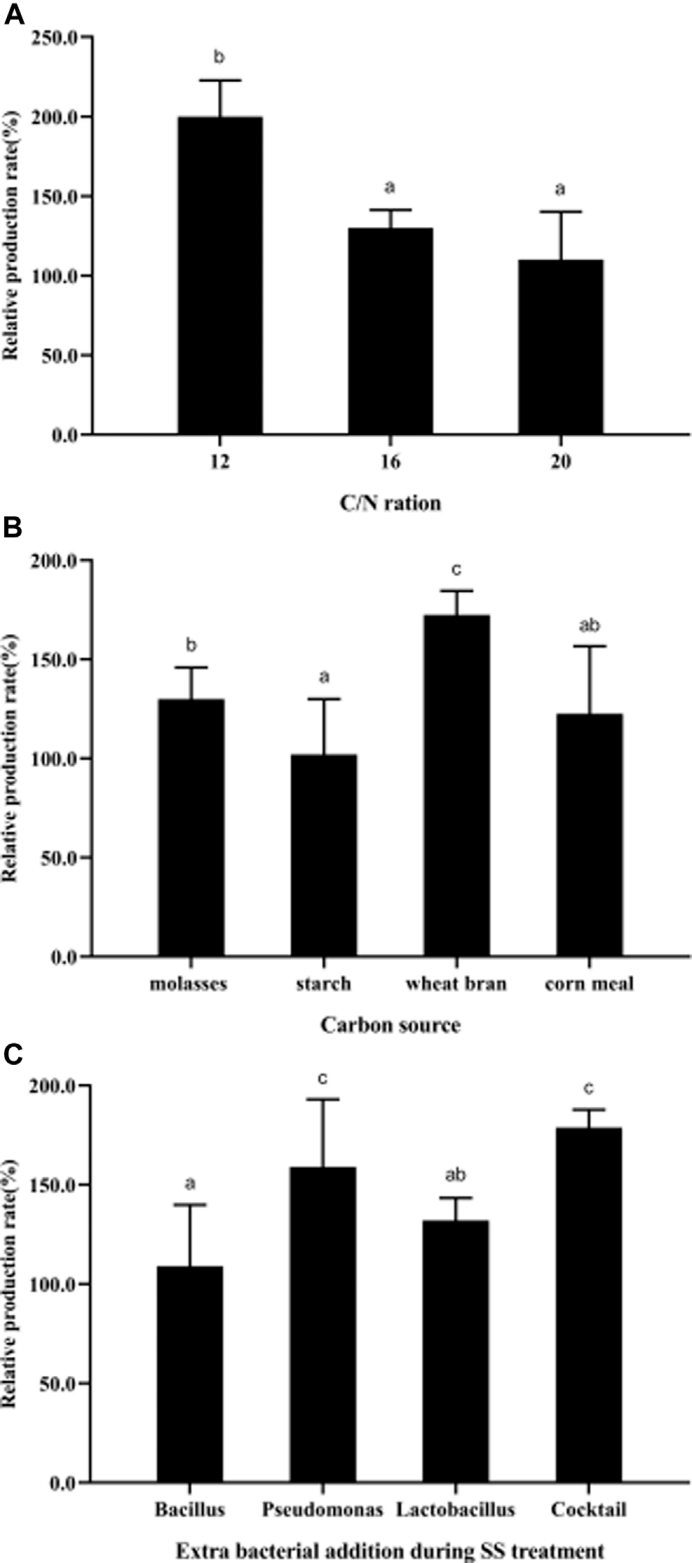
Parametric optimization for high-yield PHB production during SS treatment. **(A)**, C/N ration; **(B)**, carbon sources; **(C)**, probiotics widely used in practical aquaculture. All the data are presented as mean ± SE. Values marked with different small letters indicates significant differences among groups (*p* < 0.05). The strain information used in this study is: *Bacillus*, *Bacillus subtilis*; *Pseudomonas*, *Pseudomonas putida*; Cocktail, cocktail of *Pseudomonas* and *Lactobacillus*.

To improve PHB production, the effects of cultivation conditions (C/N ratio, carbon sources, and probiotic addition) during the treatment process were studied. The results showed that more PHB could be produced in SS at a C/N ratio of 12 with wheat bran as the carbon source, and the bacterial addition of a cocktail of *Pseudomonas* and *Lactobacillus*, through which the relative production rate could be improved by ∼200% (Figure 6).

### 3.5 Benefit Evaluation of Poly-β-Hydroxybutyrate-Enriched Suspended Solids in Aquaculture System

#### 3.5.1 Poly-β-Hydroxybutyrate-Enriched Suspended Solids Improved TAN Removal From Suspended Solids

The remediation rate in the aquaculture effluents for TAN was more than 87%. The remediation rates in SS collected from the indoor concrete tank 1 (cultured gibel carp), indoor concrete tank 2 (cultured gibel carp), indoor concrete tank 3 (cultured shrimp), outdoor pond 1 (cultured gibel carp), outdoor pond 2 (polyculture of freshwater fish), and outdoor pond 3 (cultured shrimp), were 87.19 ± 5.73%, 91.11 ± 8.94%, 87.76 ± 6.27%, 88.55 ± 3.86%, 90.17 ± 7.49%, and 80.06 ± 6.31%, respectively (Table 2). The final TAN concentration after treatment was below the tolerance range of fish or shrimp.

**TABLE 2:** Effect of the PHB-enriched treatment on total ammonia nitrogen (TAN) removal rate from suspended solids (SS).

SS samplings	Initial concentration of TAN (mg L^−1^)	Final concentration of TAN (mg L^−1^)	Removal (Mean, %)
SS from indoor concrete tank 1 (gibel carp)	16.7	2.14	87.19
SS from indoor concrete tank 2 (gibel carp)	19.9	1.77	91.11
SS from indoor concrete tank 3 (shrimp)	21.4	2.62	87.76
SS from outdoor pond 1 (gibel carp)	11.7	1.34	88.55
SS from outdoor pond 2 (polyculture of freshwater fish species)	18.3	1.7	90.17
SS from outdoor pond 3 (shrimp)	13.8	1.51	89.06

#### 3.5.2 Poly-β-Hydroxybutyrate-Enriched Suspended Soilds Promoted Fish Growth

The content of crude protein, crude lipid, ash, and total amino acids in basal SS was 29.84% (percentage of dry matter), 3.16%, 19.09%, and 14.96%, respectively. The PHB contents in the untreated SS and PHB-enriched SS were 7.06 ± 1.76% and 17.34 ± 4.76%, respectively (Supplementary Table S2).

The WG and SGR of gibel carp in the PHB-enriched SSA and USSA groups were significantly higher than those of the NSSA group. SS could be up-taken by gibel carp as a food source from water, and PHB-enriched SS had a greater effect on the growth performance. The WG, SGR, and survival rate in the PHB-enriched SSA group were 39.15%, 1.66% d^−1^, and 100%, while those in the NSSA group were -50.38%,-1.36% d^−1^ and 50%, respectively (Table 3). In the USSA group, the survival rate (23.3 ± 0.03%) was lower, possibly owing to the higher concentration of NH_4_^+^–N and NO_2_^−^–N, and non-feeding in the untreated SS.

**TABLE 3:** Growth performance of gibel carp (*Carassius auratus gibelio*) at day 30 after fed with PHB-enriched suspended solids.

Items	Groups		
	PHB-enriched suspended solid addition	Untreated suspended solid addition	No suspended solid addition
Initial weight (g)	12.37±1.45	11.77±1.48	11.94±1.03
Final weight (g)	20.33±3.18^c^	13.39±2.72^b^	7.94±1.16^a^
WG (%)	39.15±12.93^c^	12.10±2.36^b^	−50.38±3.46^a^
SGR (% day^−1^)	1.66±0.37^c^	0.43±0.12^b^	−1.36±0.07^a^
TGC	0.56±0.14^c^	0.13±0.08^b^	−0.39±0.05^a^
Survival rate (%)	100.00±7.37^c^	31.67±3.33^a^	50.00±3.85^b^

The data (mean ± standard deviation) were calculated from quadruplicate tanks, and analyzed through Duncan’s multiple range test using one-way ANOVA with SPSS software (version 24.0).

WG, weight gain; SGR, special growth rate; TGC, thermal growth coefficient.

Values marked with a different superscript for the same row are significantly different among groups (*p* < 0.05).

#### 3.5.3 Poly-β-Hydroxybutyrate-Enriched SS Up-Regulated the Immune-Related Gene Expression in Fish

**FIGURE 7:**
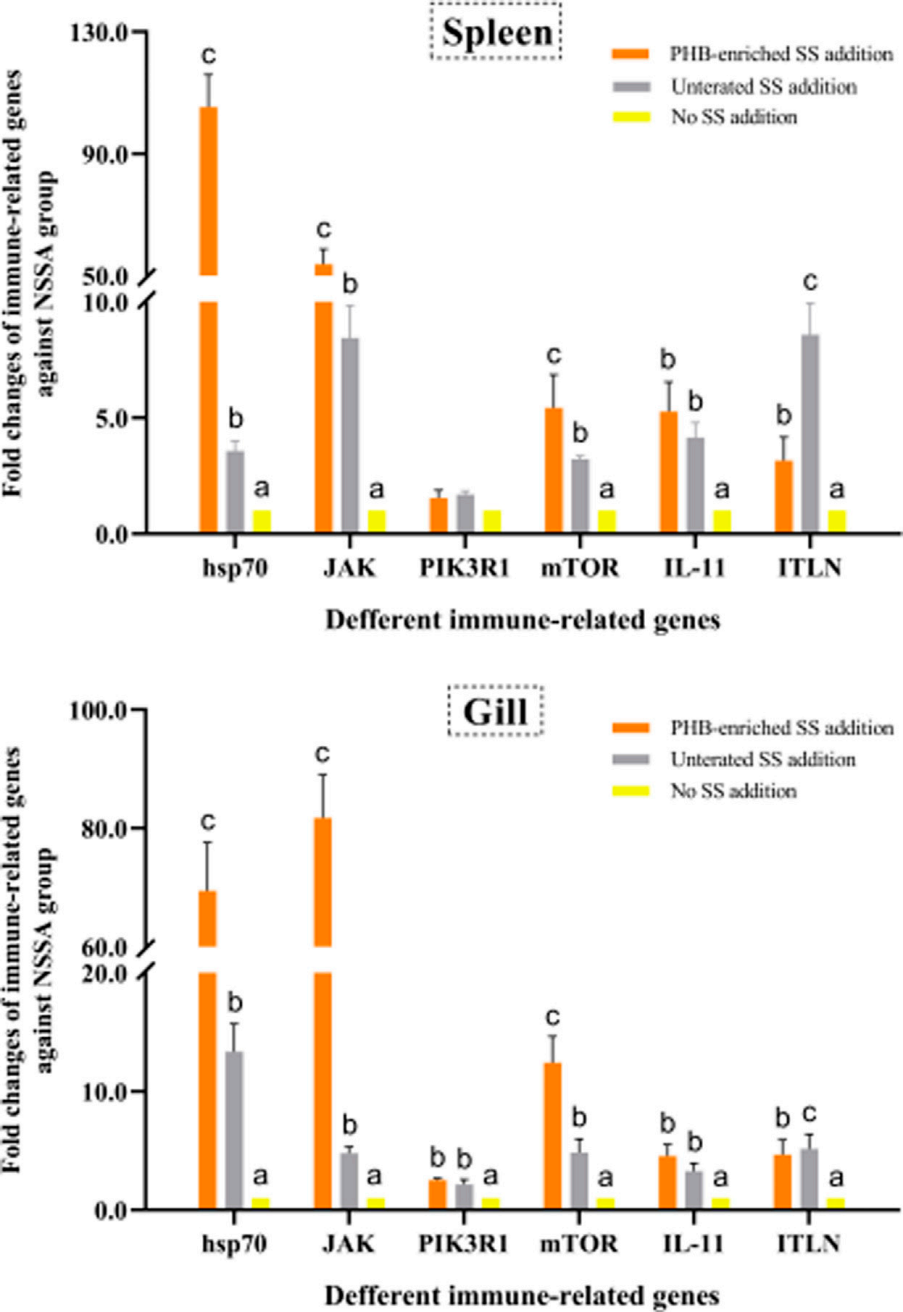
Effects of PHB-IRenriched and untreated SS addition in culture water on the immune-related gene expression in the spleen and gills of gibel carp (*Carassius auratus gibelio*).

We fed gibel carp with the PHB-enriched SS and examined the immune-related gene expression. The untreated SS were used as the control. The data showed that SS supplementation could up-regulate mRNA expression of the six immune-related genes. In particular, the expression levels of genes *hsp70*, *JAK*, and *mTOR* in both the gills and spleen were up-regulated in the PHB-enriched SSA group. Compared to the negative control NSSA group, the expressions of *hsp70* and *JAK* in the spleen from the PHB-enriched SSA group were significantly up-regulated by 105.36-fold and 53.79-fold, respectively. The mRNA expressions in the gills of the experimental group were 69.45-fold and 81.74-fold of that in the control group. The transcriptional levels of the genes in the survival fish of the USSA group were higher than those in the NSSA group, but lower than those in the PHB-enriched SSA group (Figure 7).

## 4 Discussion

PHB exists in SS from both the traditional aquaculture system and zero-water exchange system modulated by the carbon source addition. In the traditional aquaculture system, the content of pure PHB in the untreated basal SS was lower than the content of crude PHB from SS with carbon source addition during the aquaculture process (Ruan et al., 2011). GC–MS analysis showed that the retention time of our purified PHB was 2.214 min, while the PHB standard (Sigma) was 2.189 min and PHB from *Achromobacter marplatensis* was 2.21 min (Pol et al., 2017). In the infrared spectrum, the purified PHB from SS had an obvious band at 1724 cm^−1^ which was the absorption peak of hydroxyl in carboxylic acid. A series of bands at 2,850 cm^−1^, 3,436 cm^−1^, 2,976 cm^−1^, and 2,933 cm^−1^, were observed, corresponding to the stretching vibration of the -CH, -CO, -CH_3_, and -CH_2_, which were the typical characteristics of PHB. We also observed a large amount of C–O vibration between 1,057 cm^−1^ and 1,380 cm^−1^, which is consistent with the results reported by Rodríguez-Contreras et al. (2017). These data confirmed that SS from the traditional aquaculture system contained PHB and SS has the potential for PHB production.

PHB, as an intracellular energy store, is synthesized by microbes, and PHB yield is closely related to the C/N ratio, carbon sources, and bacterial diversity (Crab et al., 2012; Qiao et al., 2018; Amadu et al., 2021). Thus, the primary bacterial diversity in SS associated with PHB accumulation was analyzed, and then the accumulation condition optimized. The microbial community in SS, activated sludge, and aquaculture wastes is more complex than a single bacterial medium. The predominant phyla in SS from this study were Proteobacteria, Saprospirae, and Flavobacteriia. The predominant genera were *Comamonas*, Xanthomonadaceae_unclassified, *Acinetobacter*, Saprospiraceae_unclassified, Xanthobacteraceae_unclassified, *Wautersiella*, *Pseudomonas* and *Myxococcales*_unclassified. Similarly, in the activated sludge, the main functional microorganisms are phosphorus accumulating organisms (0.43%–5.34%) and glycogen accumulating organisms (0.16%–10.08%). Eight microorganisms were predicted as major PHB accumulators (Ju et al., 2008; Wang et al., 2013). Proteobacteria, Acidobacteria, and Burkholderiales were the dominant bacterial population associated with PHB production in the activated sludge (Wang et al., 2013; Miao et al., 2016), and the genera related to denitrification were identified as *Paracoccus*, *Ottowia*, *Theresa*, and Comamonadaceae_unclassified, accounting for 46.21% of total bacteria. Thus, *Proteobacteria*, *Comamonas*, *Acinetobacter*, and *Pseudomonas* in SS are potentially related to PHB production. Similar results were also reported in wastewater and activated sludge (Ju et al., 2008; Wang et al., 2013; Miao et al., 2016). Based on the bacteria isolated from SS using the Sudan Black B staining method (data not shown), and probiotics used in the aquaculture practice, the effects of bacterial strains including *Bacillus* sp., *Pseudomonas* sp., and *Lactobacillus* sp. on PHB accumulation in SS were studied. The results showed that *Pseudomonas* sp. and *Lactobacillus* sp. had more effective improvement in PHB production.

The C/N ratio and carbon sources are the two key factors in stimulating SS formation and PHB accumulation (Avnimelech, 1999; Fontenot et al., 2007; Albuquerque et al., 2010; Amadu et al., 2021). In the present study, we recommended the optimal C/N ratio is 12:1, which is slightly higher than that in shrimp cultured wastewater (C/N ratio of 10:1) (Fontenot et al., 2007), but much lower than that of previous studies which did not add additional bacteria supplementation in water (Wu et al., 2016; Alsafadi & Al-Mashaqbeh, 2017; Pol et al., 2017; Rodríguez-Contreras et al., 2017). Also, the cost of organic carbon can directly affect the economic profitability of culture industry (Kiani et al., 2020; Iber et al., 2021). Wheat bran as a carbon source promoted more PHB production than molasses, starch, and corn meal. These four kinds of carbon resources can significantly reduce the cultural costs (Solaiman et al., 2006). Previous studies showed that olive mill wastewater could be used as the sole carbon source to produce PHAs by *Haloferax mediterranei* (Alsafadi & Al-Mashaqbeh, 2017). In solid-state fermentation, *Cupriavidus necator* cultured in the medium with soy cake or soy cake with sugarcane molasses could produce PHB (Oliveira et al., 2007; Sharma et al., 2016). In pure bacterial culture, the carbon source significantly affects PHB production by *Pseudomonas* sp. (Matsusaki et al., 1998), *Bacillus aryabhattai* (Balakrishna Pillai et al., 2017), and *C. necator* (Sharma et al., 2016). The maximum PHB production by *B. aryabhattai* was obtained when glucose was used as the sole carbon source, rather than fructose, maltose, starch, or glycerol (Balakrishna Pillai et al., 2017). Taken together, we recommended the C/N ratio of 12, wheat bran as carbon source, and mixed bacterial addition of *Pseudomonas* sp. and *Lactobacillus* sp., which could improve the PHB accumulation during SS treatment.

PHB enrichment in SS could eliminate TAN efficiently. The nitrogen in SS is one of detrimental elements in aquaculture systems and the main components of fish fecal droppings (Lazzari & Baldisserotto, 2008; Lananan et al., 2014; Dauda et al., 2019; Rommozzi et al., 2020). In this study, the TAN removal rate of PHB enrichment was more than 87%, which is higher than bioremediation treatment in shrimp culture (68.53%) (Tsukuda et al., 2015), but lower than microalga treatment (100%) (Viegas et al., 2021). As reported previously, for hydroponic bio-filtration, TAN removal was 0.18–0.22 g m^−2^ d, TN removal was 2.64–4.32 g m^−2^ d; for sand biofiltration, TN in a rainbow trout (*Oncorhynchus mykiss*) culture system was reduced from 17.52 mg L^−1^–14.11 mg L^−1^. For biofloc technology, the removal of TAN in shrimp and mullet culture systems was 0.12–0.17 mg L^−1^, which was safe for the aquaculture species. TAN can be determined by measuring the ammonia and ammonium ions (Dauda et al., 2019; Kır et al., 2019).

Although PHB is an eco-friendly material and can be degraded into CO_2_ and H_2_O, whether the treated SS (PHB-enriched SS) has the potential to be recycled and reused in the culture system still remains unclear. Previous reports demonstrated that PHB supplementation in diet and culture water can improve the growth, immunity, and disease resistance of aquatic animals, such as tilapia (*Oreochromis mossambicus*), soiny mullet (*Liza haematocheila*), Pacific white shrimp (*Litopenaeus vannamei*), and giant river prawn (*Macrobrachium rosenbergii*) (Nhan et al., 2010; Schryver et al., 2010; Deng et al., 2014; Xu et al., 2014; Meirong et al., 2018; Qiao et al., 2019a, 2019b, 2020). Dietary PHB supplementation can up-regulate the mRNA expression of some immune-related genes such as *Hsp70*, *MHCs*, *TLRs*, *IL-8*, *hepcidin*, *pbpA*, and *AOX* in fish (Qiao et al., 2019a, 2020). Additionally, PHB can enhance the disease resistance of aquatic animals against both bacterial and viral infections (Deng et al., 2014; Defoirdt et al., 2018; Van Hung et al., 2019; Qiao et al., 2020). In the present study, PHB-enriched SS can be up-taken by gibel carp, which is in accordance with the previous studies which confirmed that SS can be up-taken by omnivorous fish and shrimp (Nhan et al., 2010; Meirong et al., 2018; Wu et al., 2018; Zhang et al., 2018). The nutritional composition of SS was similar to that composition of SS obtained from the tilapia and shrimp BFT system (Luo et al., 2014; Rajkumar et al., 2016), which can meet the demands of fish and shrimp (Luo et al., 2014; Rajkumar et al., 2016; Zhang et al., 2018). Compared with the untreated SS, the PHB-enriched SS (more than 2-fold) showed positive effects on fish growth and immunity. The WG, SGR, and survival rate of fish cultured in water that contained PHB-enriched SS were higher than those of untreated SS and no SS supplementation groups without feeding, which might be more related to higher PHB accumulation, rather than the probiotics stimulation. Accordingly, it was reported that PHB-accumulating bacteria can improve the survival and growth of shrimp, artemia, and fish more effectively than single probiotics (Gao et al., 2018; Laranja et al., 2018; Krasaesueb et al., 2019). The WG (39.15%) and SGR (1.66% day^−1^) in the PHB-enriched SS group were higher than those in the bacterial single treatment group, which were 20.46% and 0.40% day^−1^, respectively (Xu, 2019). It suggested that ‘PHB + probiotics’ treatment is much better than single probiotic usage in fish culture. The lowest survival rate of gibel carp in the untreated SS group without feeding was observed, which might be caused by higher NO_2_^−^ concentration and non-feeding. NO_2_^−^ is an intermediate toxic compound, which affects the hemoglobin in carrying oxygen and threatens to destabilize electrolytes (Jiang et al., 2014). As a result, the higher concentration of NO_2_^−^ in water causes stress, weakens growth, damages the internal organs, and reduces disease tolerance of aquatic animals, and thus harms aquaculture farming (Hurtado et al., 2016). A lower survival rate (50.00 ± 3.85%) in the no SS addition group was also observed, which is considered to relate to the 1 month non-feeding as reported by Zhang et al. (2018). The 1 month survival rate of gibel carp (41.02 ± 3.2 g) in the non-feeding freshwater culture group was 50.00 ± 7.37% (Zhang et al., 2018). PHB-enriched SS could up-regulate the expressions of the immune-related genes, such as *hsp70*, *ITLN*, and *JAK* in the spleen and gills of gibel carp. The spleen is an immune organ of fish (Li et al., 2014), and the gills are one of the first organs directly exposed to the water environment, and are considered as a major organ related to the mucosal immunity (Murray et al., 2007; Andrews et al., 2010). The *hsp70*, *ITLN*, and *JAK* play important roles in the immune response of gibel carp against both viral and bacterial infections (Podok et al., 2014a, 2014b; Xu et al., 2014). Hsp70, as a molecular chaperone, plays very important roles in anti-oxidation, anti-apoptosis, cellular immunity, and innate immune response (Wachstein et al., 2012). The expression of ITLN in gibel carp fed only with bioflocs at a TSS concentration of 600 mg L^−1^ for 30 days was up-regulated by 78.1-fold (Zhang et al., 2018). The present study would provide a better food source to aquatic animals, and greatly simplify the addition process of PHB in diets due to its water insolubility and reduction of the costs of commercial products.

Thus, from an ecological point of view, PHB can be enriched in SS to reduce nitrogen emission and be non-toxic to the environment, since polymer PHB is naturally degraded into CO_2_ and H_2_O. Notedly, this study provides a novel method for using SS in aquaculture systems to reduce waste emission. From an economic point of view, PHB acclimation is derived from low-cost raw materials, and PHB-enriched SS can be reused as a food source to promote fish growth and immunity. It provides cheaper food, and a simple and feasible approach to apply PHB in aquaculture.

## 5 Conclusion

PHB exists in SS from aquaculture system. PHB accumulation in SS is related to Proteobacteria, *Comamonas*, *Acinetobacter*, and *Pseudomonas*. Optimizing the carbon source and microbial addition increased PHB production in SS. The PHB-enrichment SS reduced TAN, improved fish growth, and boosted fish immunity. We studied SS waste in the aquaculture system and developed an approach to transforming SS waste into high-quality fish food.

## Data Availability

The original contributions presented in the study are included in the article/Supplementary Material, further inquiries can be directed to the corresponding authors.
