# Supplementary material for: An Eco-Friendly Conversion of Aquaculture Suspended Solid Wastes Into High-Quality Fish Food by Improving Poly-β-Hydroxybutyrate Production
Source: Front Physiol. 2022 May 26;13:797625. doi: 10.3389/fphys.2022.797625 (PMC9205610; doi:10.3389/fphys.2022.797625)
Supplement: Supplementary file 1 [file DataSheet1.docx]

**SUPPLEMENTARY TABLE 1** The primer sequences of six immune-related genes in gibel carp (*Carassius auratus gibelio*)

| **Gene name** | **Primer sequence (5′-3′)** |
| --- | --- |
| hsp70-F | TACACGTCCATCACCAGAGCGC |
| hsp70-R | CCCTGCCGTTGAAGAAATCCT |
| JAK-F | CGCTCTCCGTGTAGACCTGATCC |
| JAK-R | GTGGCAGCGGTGAGCAAGTG |
| PIK3R1-F | AACATTAACCGCATCCAGGCAGAG |
| PIK3R1-R | TGACCTCACCATCCACAACAACAC |
| mTOR-F | GAGAGGCCTGTGTCAAGCAA |
| mTOR-R | GACGAGCCACAGACAAGTGG |
| IL-11-F | AGAGAGACGAACTTTGGAGCACG |
| IL-11-R | GAGGAGTCACCCAGCAATTTCATAT |
| ITLN-F | CGCTTCATTTTCTTCCTGATGTGTC |
| ITLN-R | TTCTGGGTTTATGTAGGTGCCATTTAT |

F, forword primer; R, reverse primer; hsp70, heat shock protein 7; JAK, tyrosine-protein kinase; PIK3R1, phosphatidylinositol 3-kinase regulatory subunit alpha; mTOR, serine/threonine- protein kinase Mtor; IL11: Interleukin11; ITLN1: Intelectin1.

**SUPPLEMENTARY TABLE 2** Proximate composition (as a percentage of dry matter, n=3) of suspended solids (SS)

| **Proximate composition** | **SS** | **PHB-enriched SS** | **Note** |
| --- | --- | --- | --- |
| Crude protein | 29.84±3.62 | ND | Zhang et al., 2018 |
| Crude lipid | 3.16±0.64 | ND | Zhang et al., 2018 |
| Ash | 19.09±0.41 | ND | Zhang et al., 2018 |
| Total amino acids | 14.96 | ND | Zhang et al., 2018 |
| PHB content | 7.06±1.76 | 17.34±4.75 | This study |

SS were collected from the indoor concrete tank cultured gible carp. Values are shown as mean±SD. PHB, poly-β-hydroxybutyrate. ND, not detected.


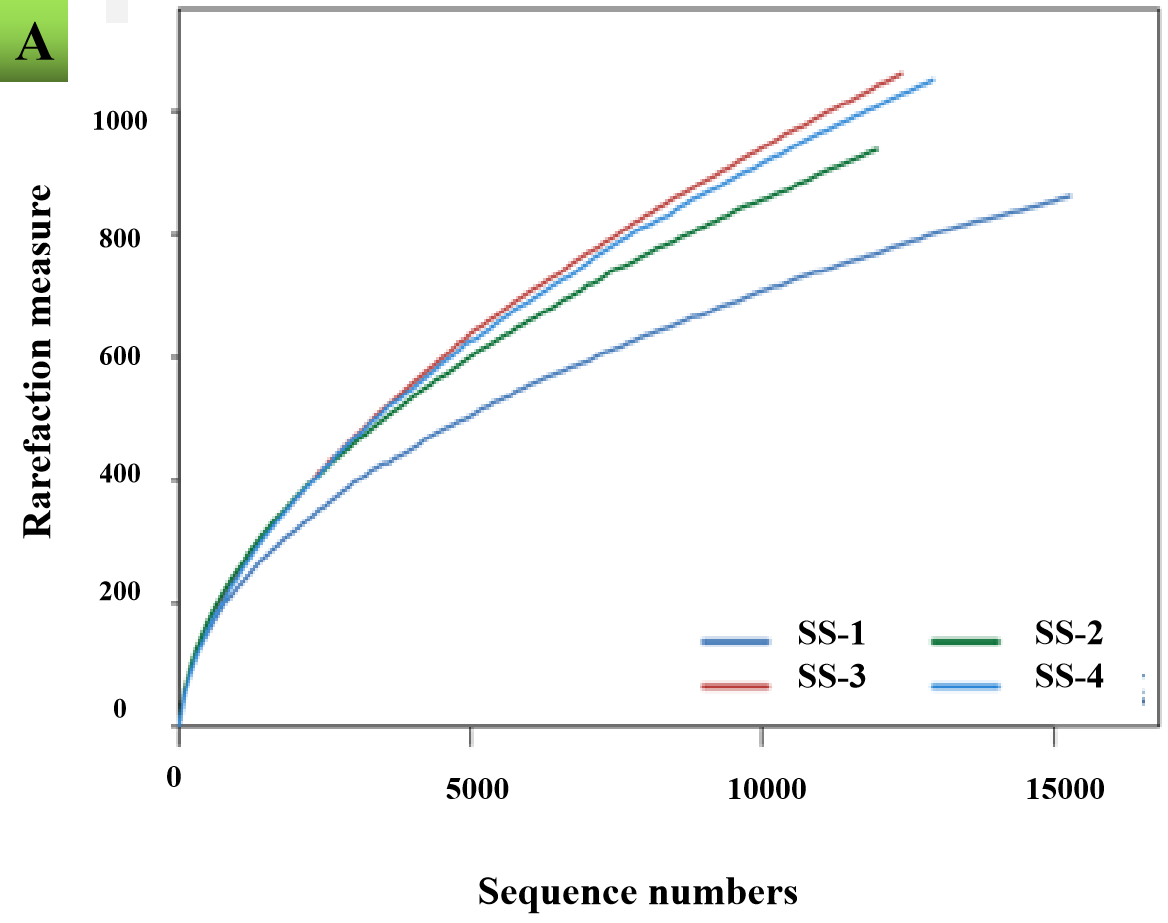


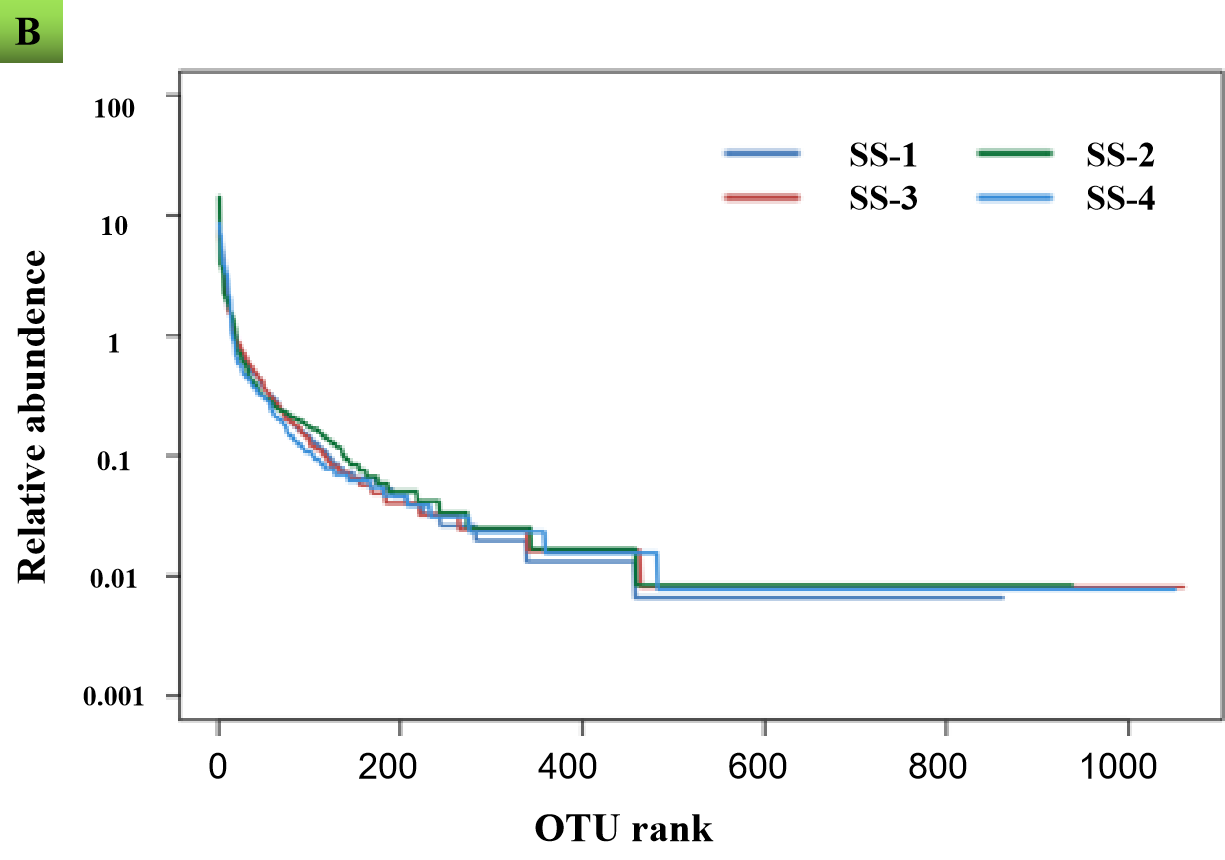


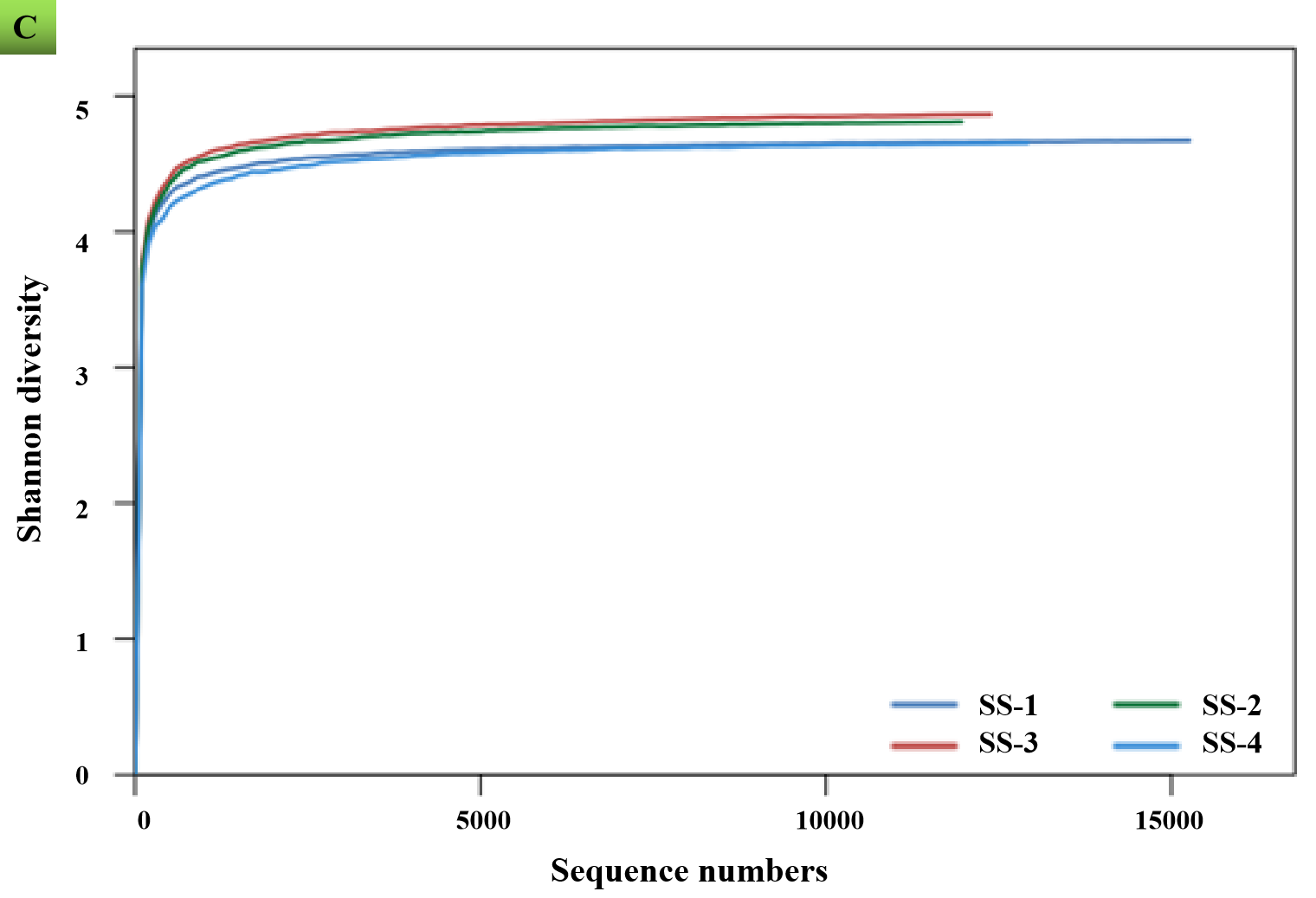


**SUPPLEMENTARY FIGURE 1 |** Rarefaction curve (A), relative abundance (B) and Shannon diversity (C) of operation taxonomic unites (OUTs) from quadruple SS samples.
